# Mitigation Effect of Dense “Water Network” on Heavy PM_2.5_ Pollution: A Case Model of the Twain-Hu Basin, Central China

**DOI:** 10.3390/toxics11020169

**Published:** 2023-02-10

**Authors:** Yan Zhu, Yongqing Bai, Jie Xiong, Tianliang Zhao, Jiaping Xu, Yue Zhou, Kai Meng, Chengzhen Meng, Xiaoyun Sun, Weiyang Hu

**Affiliations:** 1 Hubei Meteorological Service Center, Wuhan 430205, China; 2 China Meteorological Administration Basin Heavy Rainfall Key Laboratory/Hubei Key Laboratory for Heavy Rain Monitoring and Warning Research, Institute of Heavy Rain, China Meteorological Administration, Wuhan 430205, China; 3 Key Laboratory for Aerosol-Cloud-Precipitation of China Meteorological Administration, Collaborative Innovation Center on Forecast and Evaluation of Meteorological Disasters, Nanjing University of Information Science and Technology, Nanjing 210044, China; 4 Jiangsu Climate Center, Nanjing 210044, China; 5 Key Laboratory of Meteorology and Ecological Environment of Hebei Province, Hebei Provincial Institute of Meteorological Sciences, Shijiazhuang 050021, China; 6 State Key Laboratory of Pollution Control and Resource Reuse and School of the Environment, Nanjing University, Nanjing 210023, China

**Keywords:** air pollution, PM_2.5_, “water network”, meteorological drivers, the Weather Research and Forecasting Model with Chemistry (WRF-Chem)

## Abstract

The influence of the underlying surface on the atmospheric environment over rivers and lakes is not fully understood. To improve our understanding, this study targeted the Twain-Hu Basin (THB) in central China, with a unique underlying surface comprising a dense “water network” over rivers and lakes. In this study, the Weather Research and Forecasting Model with Chemistry (WRF-Chem) was used to simulate the impact of this dense “water network” on a wintertime heavy PM_2.5_ pollution event in the THB. On this basis, the regulating effects of density and area of the lake groups, with centralized big lakes (CBLs) and discrete small lakes (DSLs), on PM_2.5_ concentrations over the underlying surface of the dense “water network” in the THB were clarified, and the relative contributions of thermal factors and water vapor factors in the atmospheric boundary layer to the variation of PM_2.5_ concentrations were evaluated. The results show that the underlying surface of dense “water networks” in the THB generally decreases the PM_2.5_ concentrations, but the influences of different lake-group types are not uniform in spatial distribution. The CBLs can reduce the PM_2.5_ concentrations over the lake and its surroundings by 4.90–17.68% during the day and night. The ability of DSLs in reducing PM_2.5_ pollution is relatively weak, with the reversed contribution between −5.63% and 1.56%. Thermal factors and water vapor–related factors are the key meteorological drivers affecting the variation of PM_2.5_ concentrations over the underlying surface of dense “water networks”. The warming and humidification effects of such underlying surfaces contribute positively and negatively to the “purification” of air pollution, respectively. The relative contributions of thermal factors and water vapor–related factors are 52.48% and 43.91% for CBLs and 65.96% and 27.31% for DSLs, respectively. The “purification” effect of the underlying surface with a dense “water network” in the THB on regional air pollution highlights the importance of environmental protection of inland rivers and lakes in regional environmental governance. In further studies on the atmospheric environment, long-term studies are necessary, including fine measurements in terms of meteorology and the environment and more comprehensive simulations under different scenarios.

## 1. Introduction

The underlying surface of river and lake systems is an ecosystem dominated by hydrological processes, and the water–heat exchange between water and atmosphere is an important mechanism affecting the atmospheric boundary layer and physicochemical processes of aerosols and has important implications for regional weather, climate, and the environment [1,2,3,4,5,6].

The underlying surface of “water networks” has remarkable influences on the vertical structure of temperature and humidity in the atmosphere and on the physical processes in the atmospheric boundary layer [7,8]. Compared with other types of underlying surfaces, water bodies have greater heat capacity and thermal inertia, which causes slower warming during the day and less sensible heat exchange between water and atmosphere [9]. At the same time, more latent heat is released over the water surface due to evaporation. The inhomogeneous properties of land and water surface lead to thermal differences between the two types of surfaces and thus change the thermal radiation. The changes of thermal radiation and heat exchange intensity directly affect the process of heat transfer near the surface, which further change the structure of temperature and humidity in the surrounding areas and cause changes in the structure of the local atmospheric boundary layer [10]. The temperature and humidity structures in the atmospheric boundary layer are important factors for the accumulation of air particulate matter and affect the diffusion and deposition of air pollutants [11,12,13,14,15]. The local circulation of lake–land breeze driven by heat factors will change the temperature and wind fields and other meteorological conditions in the surrounding areas, which also affects the diffusion and distribution of air pollutants [5,16,17,18,19]. During the day, under the circulation of lake breeze, air pollutant particles are transported to the land and diffused aloft by the updraft of lake breeze. The lake breeze circulation can also cause air pollutant deposition on the lakeshore, where the pollutants maintain a high concentration along the lakeshore for a long time [20]. At night, the concentration of air particulate matter over the land decreases. Under the joint influence of environmental wind and land breeze, the air particulate matter accumulates over the lake region [21]. In addition, urban rivers are conducive to the generation of secondary aerosols, which are potential sources of atmospheric organic matter [22]. At the same time, the water surface is favorable for the hygroscopic growth of fine particles in the atmosphere and hence affects the distribution of air pollutants, which thus plays a crucial role in the distribution and diffusion of regional atmospheric particulate matter [23,24,25].

The evolution process of air pollution can be fully understood by using life cycle assessment (LCA) [26] and the Weather Research and Forecasting Model with Chemistry (WRF-Chem) [27]. The WRF-Chem model (version 3.9.1) developed by the National Oceanic and Atmospheric Administration (NOAA) and National Center was used to simulate a heavy pollution event in the Twain-Hu Basin (THB). This model can couple complex physical and chemical processes such as transport, sedimentation, emissions, chemical conversions, aerosol effect, photolysis, and radiation of chemical substances and has been widely used in the simulation of air pollution [28,29,30,31].

The Twain-Hu Basin (THB), located in the middle reaches of the Yangtze River Basin of central China, has typical characteristics of a monsoon climate. It is a region with abundant water resources in river and lake systems. The area of water bodies such as rivers and lakes account for more than 20% of the plain area in the THB, which is the region with the largest share of water area in China [32]. The dense river network and numerous lakes make the THB region a typical inland humid region in China and form a humid monsoon climate.

In recent years, the THB has become a region of frequent incidence of air particulate pollution in China [33,34,35]. The underlying surface of dense “water networks” in the THB makes the spatiotemporal variations of air particulate matter and the mechanisms involved more complex, requiring further exploration. However, the studies on the influences of lake water-driven thermal circulation on the local atmospheric environment have been mainly focused on coastal cities and large lakes in plateau regions [36,37,38,39,40]; there are few studies in inland areas. Given the important regulating effect of the unique underlying surface of “water networks” on the variation of regional PM_2.5_ concentrations, clarifying the influence on the regional atmospheric environment can deepen the scientific understanding of the mechanism for air pollution variation in the inland humid area of China.

This study focused on a wintertime PM_2.5_ pollution event in the THB in central China and explored the influence of dense “water networks” in the THB on the PM_2.5_ variation and the mechanism involved. The results are expected to provide a reference for the research and prevention of air pollution in inland humid areas as well as a decision-making basis for the research and protection of urban underlying surfaces. The remainder of this paper is organized as follows. Section 2 introduces the datasets and model setup used in the simulation. Section 3 presents the main results, and the conclusions are presented in Section 4.

## 2. Materials and Methods

### 2.1. Materials

In this study, a heavy PM_2.5_ pollution event induced by stagnant weather conditions in the THB during 11–24 January 2018 was selected as the research object. The Weather Research and Forecasting Model (WRF) with Chemistry (WRF-Chem) was used to conduct a series of simulations. The Final Operational Global Analysis (FNL) data provided by the National Center for Environmental Prediction (NECP)/National Center for Atmospheric Research (NCAR) was used to adopt the initial meteorological field and boundary conditions for WRF-Chem model simulations. The spatial and temporal resolutions of the FNL data are 1° × 1° and 6 h, respectively. Surface meteorological variables such as 10 m wind speed and sea level pressure were also used in this study, which are from the fifth-generation European Centre for Medium-Range Weather Forecasts reanalysis (ERA5) dataset (https://cds.climate.copernicus.eu/cdsapp#!/dataset/reanalysis-era5-land?tab=form, accessed on 7 February 2023), with the spatiotemporal resolutions being 0.25° × 0.25° and 1 h. The hourly surface observation data obtained from the National Meteorological Center (http://data.cma.cn/, accessed on 7 February 2023) were also adopted, including temperature, humidity, wind speed and direction, atmospheric pressure, and precipitation. The hourly dataset of PM_2.5_ mass concentrations was obtained from the China Air Quality Online Monitoring and Analysis Platform (https://www.aqistudy.cn/, accessed on 7 February 2023), which currently collects the PM_2.5_ concentration data from 367 cities in China, and all the data are automatically updated every hour. The gridded dataset of emission sources adopts the 2016 Multi-resolution Emission Inventory for China (MEIC; http://meicmodel.org.cn/, accessed on 7 February 2023) [41,42], which is output by month, with the spatial resolution being 0.25° × 0.25°.

This study also updated the underlying surface data for the WRF-Chem with the MODIS Land Cover Type (MCD12Q1) product. This product adopts the storage mode of sinusoidal projection and contains multiple classification schemes, which describe land cover properties derived from observations spanning a year’s input of Terra and Aqua data. The spatial resolution is 500 m. Each image has a size of 1200 × 1200 and contains 16 layers. The primary land cover scheme product data were selected for application in this study, which identified 17 land cover classes defined by the International Geosphere Biosphere Programme (IGBP), including 11 classes of vegetation cover, 3 developed and mosaicked land classes, and 3 non-vegetated land classes. We used ArcGIS to splice and transcode Land Cover Type 1 tif data products of MCD12Q1 in 2018 (simulation period). The underlying surface data are used in the WRF-Chem model after being re-defined. In addition, the image remote sensing data of the environmental and disaster monitoring and prediction satellite from the China Resources Satellite Application Center were used to show the distribution of water and land (Figure S1c).

### 2.2. Model Setting

**Figure 1:**
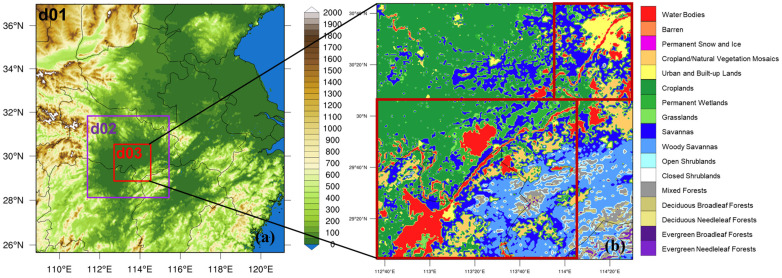
(**a**) Three nested domains d01, d02, and d03 (in different boxes) and topographical height above sea level (color contours, unit: m) for WRF-Chem simulation; (**b**) land use type in the domain d03. The big box in the lower left corner of d03 denotes the centralized big lakes (CBLs) Dongting Lake and Honghu Lake, and the small box in the upper right corner denotes the discrete small lakes (DSLs) around Wuhan.

This study employed the WRF-Chem version 3.9.1 to carry out the simulations, and the simulation period was from 20:00 10 January to 20:00 24 January 2018 (local time, the same as below). The first 24 h were used as the spin-up time, and the subsequent simulation results were used for numerical diagnosis and analysis. In this study, the center of the simulation region was (31.5° N, 115° E), and the vertical direction was divided into 38 layers. The model contained three nested regions (Figure 1). The outermost domain covers central and eastern China, with the spatial resolution being 9 km × 9 km. The middle domain includes the entire Twain-Hu Basin, with the spatial resolution being 3 km × 3 km. The inner nested region includes several small lakes scattered around Wuhan and large-lake areas of the Dongting Lake and Hong Lake, with the spatial resolution being 1 km × 1 km. The lateral boundary was updated every 6 h. The model was used in nudging the four-dimensional assimilation process, with grid analysis nudging above the planetary boundary layer, and the nudging process was applied for air temperature, wind, and air moisture, with a nudging coefficient of 3.0 × 10^−4^ s^−1^ in this study. The simulation result was output every hour.

The simulations used the Lin microphysics scheme [43], RRTM long-wave radiation scheme [44], Goddard short-wave radiation scheme [45], MM5 Monin-Obukhov near-stratum scheme, Noah land surface process scheme [46], and ACM2 planetary boundary layer scheme; the Grell 3D cumulus parameterization scheme was only applied to the coarse domain d01 with the horizontal resolution of 9 km (Figure 1a). The model was also coupled with the lake module of the community land model [47]. The results showed that the lake–atmosphere coupled model with the lake module and optimized parameters is superior to the model without the lake scheme [47,48]. The biogenic emissions were calculated through an online Model of Emissions of Gases and Aerosols from Nature (MEGAN, https://www.acom.ucar.edu/wrf-chem/download.shtml (accessed on 7 February 2023). The wintertime biological VOC emissions were much weaker than anthropogenic NMVOC emissions in the THB region over central China (Figure S2), which could indicate that anthropogenic emissions were the main sources of PM_2.5_ for the wintertime PM_2.5_ pollution event.

In order to analyze the influence of dense “water networks” on the atmospheric environment in the THB, a set of simulation experiments was performed in this study. The real land use data were used in the control experiment (denoted as lake experiment), and the water bodies in the model domain were changed to farmland underlying surface in the sensitive experiment (denoted as no-lake experiment). By studying the differences between the meteorological elements and air pollutants simulated from the lake experiment and no-lake experiment, the effect of the underlying surface with dense “water networks” in the THB was investigated as follows.

### 2.3. Methods of Statistical Analysis

Several statistical indicators, including correlation coefficient (R), root mean square error (RMSE), mean bias (MB), general error (GE), mean fractional bias (MFB), and mean fractional error (MFE) were used to evaluate the simulation results. The indicators were defined as follows:

(1) $$R=\frac{\sum_{i=1}^{N}\left(X_{i}-\bar{X}\right)\left(Y_{i}-\bar{Y}\right)}{\sqrt{\sum_{i=1}^{N}{\left(X_{i}-\bar{X}\right)}^{2}}\sqrt{\sum_{i=1}^{N}{\left(Y_{i}-\bar{Y}\right)}^{2}}},$$

(2) $$RMSE=\sqrt{\frac{\sum_{i=1}^{N}{\left(X_{i}-Y_{i}\right)}^{2}}{N}},$$

(3) $$MB=\frac{\sum_{i=1}^{N}\left(X_{i}-Y_{i}\right)}{N},$$

(4) $$GE=\frac{\sum_{i=1}^{N}\left|X_{i}-Y_{i}\right|}{N},$$

(5) $$MFB=\frac{1}{N}\sum_{i=1}^{N}\frac{\left(X_{i}-Y_{i}\right)}{\left(X_{i}/2+Y_{i}\right)}\times 100\%,$$

(6) $$MFE=\frac{1}{N}\sum_{i=1}^{N}\frac{\left|X_{i}-Y_{i}\right|}{\left(X_{i}/2+Y_{i}\right)}\times 100\%,$$

where $X_{i}$ and $Y_{i}$ represent the simulated and observed values, respectively. The indicator of R with a value closer to 1, or MB with a value closer to 0, or RMSE and GE with smaller values indicate a better simulation effect [49].

In order to quantify the effects of meteorological elements on PM_2.5_ variations induced by the underlying surface of “water networks”, multiple linear regression is adopted to quantify the contribution of meteorological factors to the change in air pollutants by using the software MATLAB (https://ww2.mathworks.cn/, accessed on 7 February 2023).

## 3. Results and Analysis

### 3.1. Simulation and Verification of Heavy Pollution Process

**Figure 2:**
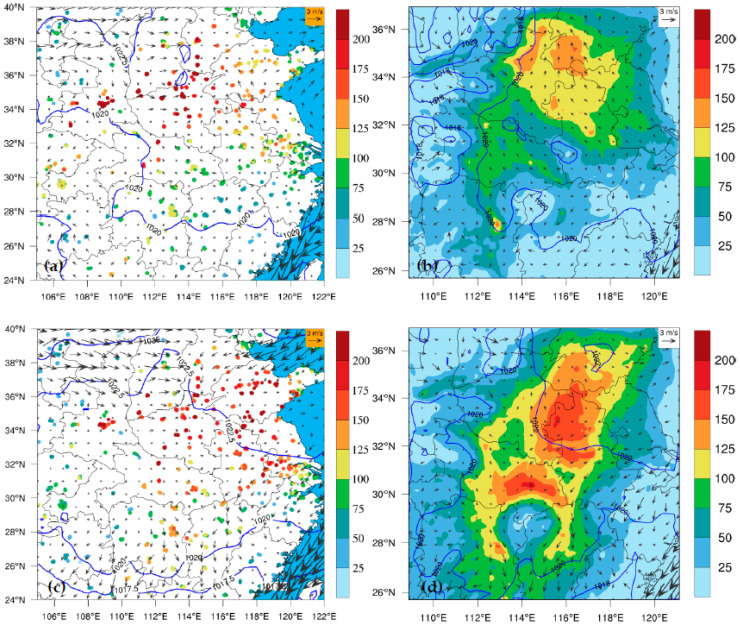
Spatial distribution of (left panel) observed and (right panel) simulated near-surface PM_2.5_ concentrations (color dots (left panel), color contours (right panel), μg/m^3^), and 10 m wind vectors (m·s^−1^) averaged (**a**,**b**) over 14–17 January and (**c**,**d**) over 19–22 January in 2018. Blue lines indicate sea level pressure (hPa). Blue area denotes the Pacific Ocean in the left panels.

From 14 to 22 January in 2018, a local heavy pollution event induced by stagnant weather conditions occurred in the THB (Figure S3). From 14 to 17 January, the local pollutants were gradually accumulated, and the air quality gradually deteriorated in the THB. Air pollution in the THB became worse on 18 January due to the pollutants transported by weak cold air from northern China. From 19 to 22 January, as the THB was controlled by the uniform pressure field after the weak cold air passed through, the wind speed was small, with poor diffusion conditions in the ambient atmosphere. Therefore, the cumulative growth of pollutants led to continuous severe PM_2.5_ pollution. Figure 2 shows the spatial distributions of observed and simulated PM_2.5_ concentrations during 14–17 January and the 10 m wind field during 19–22 January. It can be seen that the THB in both stages was affected by weak wind and stagnant weather conditions in the uniform pressure field. The model can well reproduce the continuous accumulation process of air pollutants in the THB under stagnant weather conditions. The simulations are consistent with the observations with respect to the large-value centers of PM_2.5_ concentrations in North China and the THB. We also estimated the spatial distributions of simulated PM_2.5_ concentrations and weather conditions against observations over domain 01 from 14 January to 24 January (Figure S3); the simulated wind pressure fields were reasonably close to the observed, and the simulated spatial distribution characteristics of PM_2.5_ were relatively consistent with the observations.

**Figure 3:**
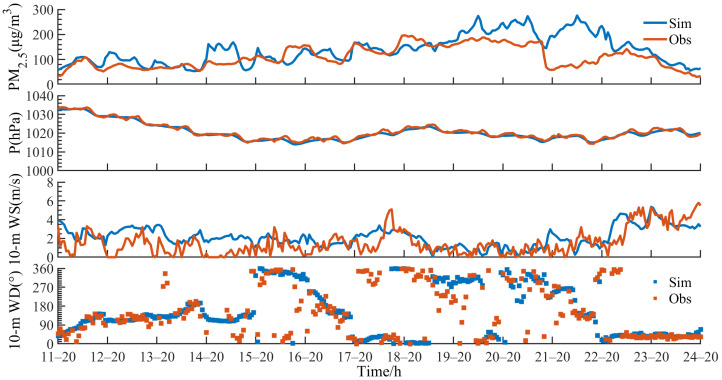
Comparison between simulation and observation of near-surface PM_2.5_ concentrations, surface air pressure (P), 10-m wind speed (10-m WS), and 10-m wind direction (10-m WD) in Wuhan from 20:00 11 January to 20:00 24 January 2018.

We used the hourly observations of 2 m air temperature, surface air pressure, 2 m humidity, 10 m wind speed, 10 m wind direction, and precipitation rate at the representative station in the inner domain d03 (Figure 3 and Table 1). Figure 3 shows the simulated and observed near-surface PM_2.5_ concentrations, surface air pressure, 10 m wind speed, and 10 m wind direction at Wuhan national meteorological observation station from 20:00 11 January to 20:00 24 January 2018. The model well describes the evolution of this pollution process and the variations of the meteorological elements. From 14 to 17 January, the wind speed is maintained at about 2 m·s^−1^, and the PM_2.5_ concentration increases slowly. On 18 January, the wind speed increases as the weak cold air moves southward, and the pollution increases due to the pollutant transport. From 19 to 22 January, the wind speed decreases remarkably to about 1 m·s^−1^, which contributes to the continuous accumulation of pollutants. On 23 January, the wind speed increases, and the pollution quickly dissipates. During the early stage of the pollution process, the simulated PM_2.5_ concentration is close to the observations. However, in the later stage, the simulations present some deviation from the observations in terms of the spatial distribution due to the underestimated precipitation and higher model resolution than that of the emission inventory. During the pollution process, the PM_2.5_ concentration is overestimated in simulations, but the changing trend is generally consistent with the observations. Boylan et al. [49] believed that the MFB and MFE are more suitable for evaluating the model performance for prediction of particulate matter and proposed that “reasonable” prediction corresponds to −60% ≤ MFB ≤ 60% and MFE ≤ 75%, and “ideal” prediction corresponds to −30% ≤ MFB ≤ 30% and MFE ≤ 50%. Table 1 shows the evaluation results for each simulated variable at Wuhan and Yueyang stations in the simulation domain d03. We also used the method mentioned in Astruop’s paper [50], with the direction differences normalized to stay within ±180 degrees to calculate the statistical parameters of wind direction (Table S1). All the values of evaluation indicators are within reasonable ranges, indicating that the simulation effect is relatively ideal, which further proves that the model has good applicability over the underlying surface of “water networks”.

### 3.2. Influence of Dense “Water Network” on PM_2.5_ Concentrations

**Figure 4:**
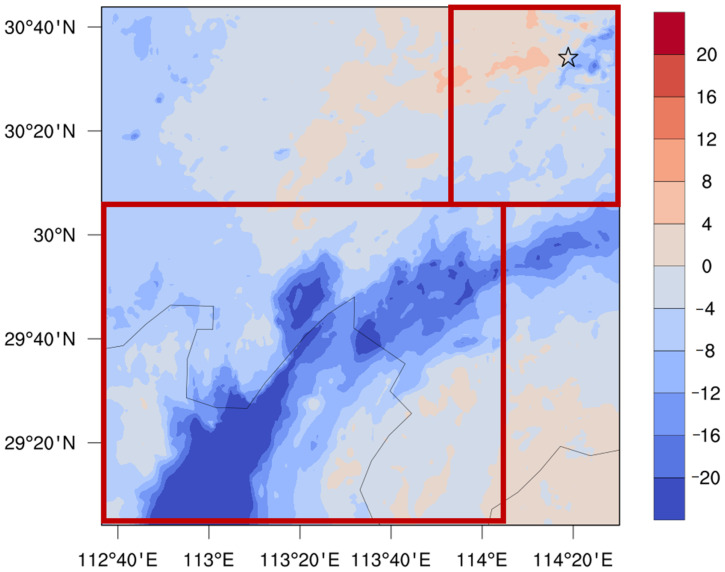
Spatial distribution of averaged PM_2.5_ concentration difference (μg/m^3^) between the lake experiment and no-lake experiment during the pollution process, 19–22 January 2018.

The simulation results show that the air pollution in the THB reaches the level of heavy PM_2.5_ pollution during 19–22 January 2018 (Figure S4). In order to clarify the regulating effect of the underlying surface of “water networks” on heavy air pollution, the influence of such underlying surfaces on the spatiotemporal distribution of PM_2.5_ concentrations in the THB during the heavy pollution process was first analyzed. The comparison between the lake experiment and no-lake experiment reveals that the underlying surface of “water networks” in the THB generally reduces the regional PM_2.5_ concentrations (Figure 4), especially over the lake and its surrounding areas. However, the influence from the underlying surface is not uniform in the spatial distribution. In areas surrounding the Dongting Lake and Honghu Lake, the PM_2.5_ concentration decreases remarkably. The PM_2.5_ concentration over the lake surface decreases by more than 20 μg/m^3^, reducing the regional average PM_2.5_ concentrations by 9.43%. However, for numerous scattered small lakes around Wuhan, the impact from the underlying surface of “water networks” on PM_2.5_ concentrations is rather complex, which is manifested as the local aggravation or alleviation of PM_2.5_ pollution, contributing negatively to the regional average PM_2.5_ concentrations by 0.79%. Compared with the land, water bodies have greater heat capacity and thermal inertia, which most directly affects the vertical water vapor flux [8]. Although the area proportions of water networks for centralized big lakes (CBLs) and discrete small lakes (DSLs) are similar (12.5% and 11.8% of total grid points for the water networks in the two types of lakes, respectively), the simulation results show that the upward fluxes of water vapor over these two types of underlying surface are very different (Figure S2). Different degrees of water-heat exchange cause differences in local meteorological conditions, which further regulates the spatial distribution of PM_2.5_ concentrations. This may be one reason for the non-uniform distribution of the impacts from the dense water network on the regional atmospheric environment in the THB.

**Figure 5:**
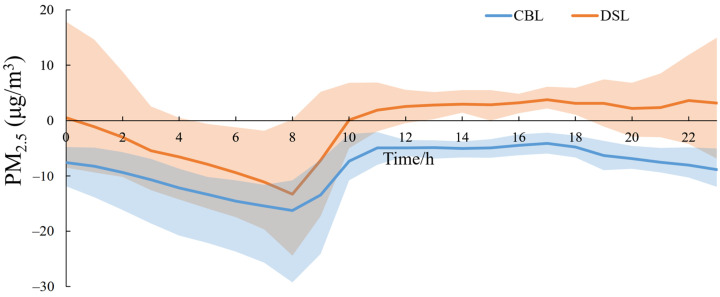
Diurnal variations of the near-surface PM_2.5_ concentration differences (μg/m^3^) between the simulations from the lake experiment and no-lake experiment, averaged over CBLs and DSLs during the air pollution process of 19–22 January 2018. The shaded regions are the extreme value intervals over CBLs and DSLs.

Figure 5 shows the diurnal variations of near-surface PM_2.5_ concentration differences between the simulations from the lake experiment and no-lake experiment averaged over CBLs and DSLs during the air pollution process. Figure S5 shows the PM_2.5_ concentrations in the lake experiment simulation. It is revealed that the influences of the “water network” on the PM_2.5_ exhibit obvious diurnal variations, which vary with the density and area of “water network”. Around sunrise, water bodies cause the greatest decreases in PM_2.5_ concentrations over both CBLs and DSLs. The CBLs reduced PM_2.5_ by an average of 16.28 μg/m^3^ at 08:00 (maximum 29.28 μg/m^3^), while the DSLs reduced PM_2.5_ by an average of 13.29 μg/m^3^ (maximum 24.42 μg/m^3^). More significantly, the CBLs reduced the PM_2.5_ concentrations all day, with the stronger potential to “purify” PM_2.5_ particles at night. The DSLs aggravated local PM_2.5_ pollution from 10:00 to 00:00, with the peak of 17.91 μg/m^3^ at 00:00 and decreasing PM_2.5_ concentrations in other periods. In general, CBLs can result in a decrease of the local PM_2.5_ concentrations by 4.15–16.28 μg/m^3^ (4.90–17.68%), while the underlying “water network” of DSLs has weak potential to clean the air environment, with the reverse impacts on PM_2.5_ levels ranging from −13.29–3.76 μg/m^3^ in the relative change rates from a negative value of −5.63% to a positive value of 1.56%.

**Figure 6:**
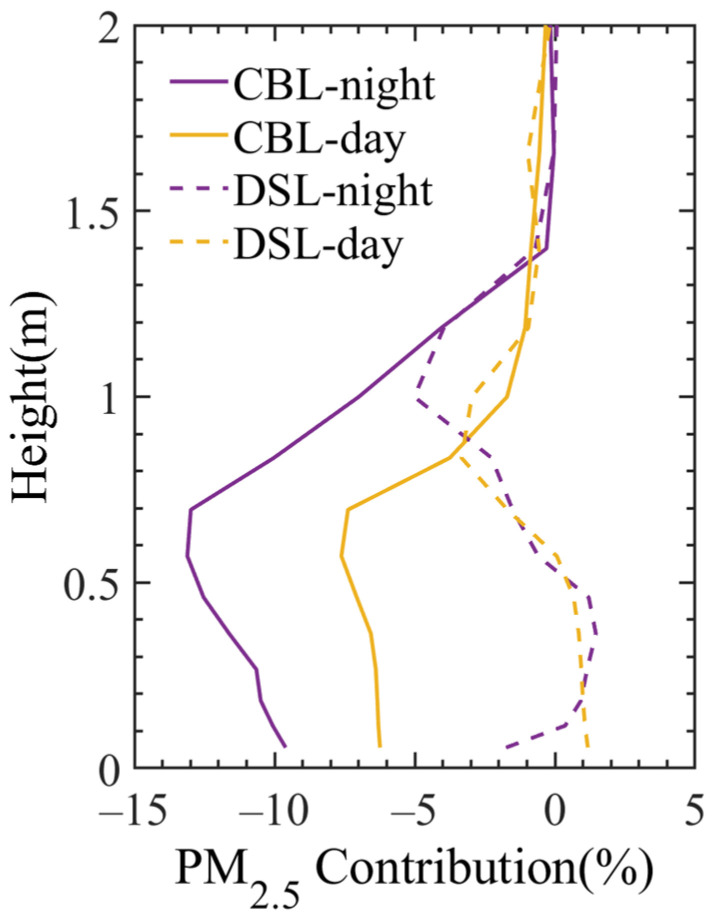
Vertical distribution of PM_2.5_ contribution differences (%) between the lake experiment and no-lake experiment averaged for CBLs and DSLs below 2 km in the atmosphere during the air pollution process of 19–22 January 2018. The yellow and purple lines represent the daytime at 14:00 (local time) and the nighttime at 02:00 (local time).

Figure 6 shows the influences of the density and area of “water networks” on the vertical distribution of PM_2.5_ concentrations below 2 km in the atmosphere. Figure S6 shows the PM_2.5_ concentrations in the lake experiment simulation. It can be seen that the CBLs improve the local air quality remarkably, which can reduce the PM_2.5_ concentrations from the ground up to 1500 m at 02:00. Moreover, the PM_2.5_ concentration within the height of 500 m above the ground can be reduced by about 9.60–13.11%. At 14:00, the PM_2.5_ concentration near the ground in the surrounding area of the CBLs decreases by 1.71–6.24%, and the influence range reaches up to 1000 m in the vertical direction. In contrast to the CBLs, the influence of DSLs on PM_2.5_ concentration is more complicated, which is manifested by greater uncertainty and weak effects near the surface. In addition, the DSLs tend to deteriorate the air quality near the surface during the daytime.

### 3.3. Influence Mechanism of Dense “Water Network” on Atmospheric Environment

The river and lake systems are usually cold in the day and warm at night. That is, the lake acts as a cold (warm) source, which has a good cooling (warming) effect in the daytime (nighttime). Meanwhile, the water vapor evaporation will cause more latent heat release. The inhomogeneity of water and land properties can affect the spatiotemporal distribution of sensible heat flux and latent heat flux [8].

**Figure 7:**
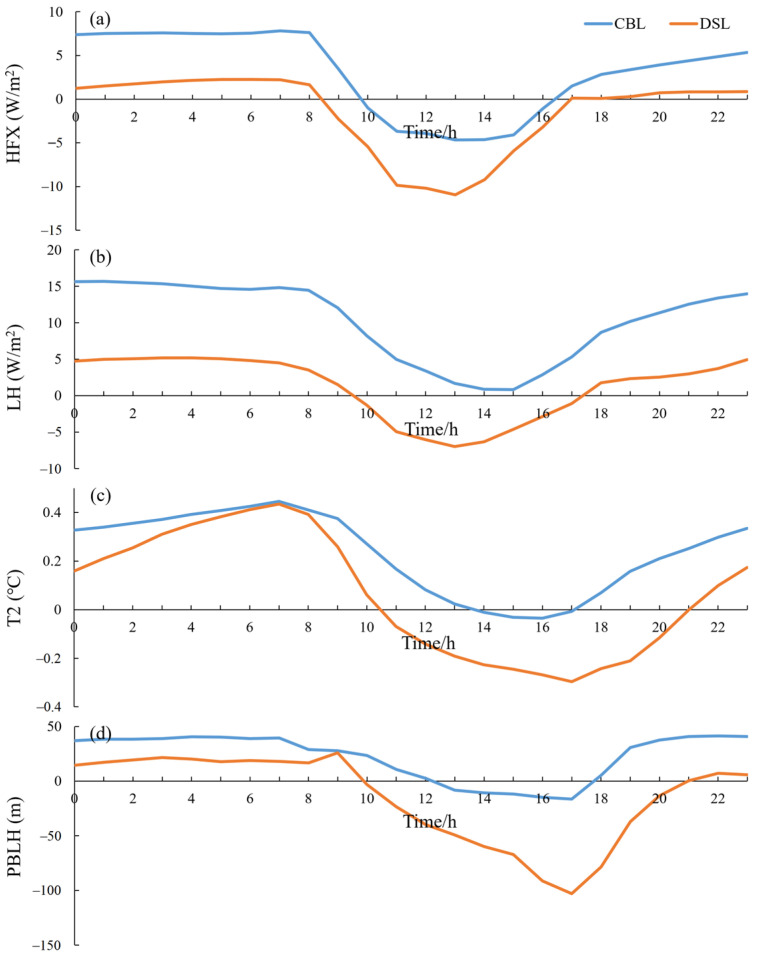
Diurnal variations in differences of (**a**) sensible heat flux (HFX, W/m^2^), (**b**) latent heat flux (LH, W/m^2^), (**c**) 2-m air temperature (T2, °C), and (**d**) planetary boundary layer height (PBLH, m) between the simulations of the lake experiment and no-lake experiment averaged for CBLs and DSLs during the air pollution process from 19–22 January 2018.

Figure 7 shows the diurnal variations for the differences of simulated, spatially averaged sensible heat flux, latent heat flux, 2 m temperature, and atmospheric boundary layer height between the lake experiment and no-lake experiment for the CBLs and DSLs. Figure S7 shows the variables in the lake experiment simulation. During the day, the sensible heat flux over the underlying surface of “water networks” is reduced, which causes little evaporation and weak variation of latent heat flux over the CBLs. The decreases of sensible and latent heat fluxes over the DSLs during the day are relatively more obvious, which make the surrounding areas of the DSLs a strong cold source. At night, the sensible and latent heat fluxes over the underlying surface of “water networks” increase remarkably, especially for the latent heat release over the CBLs. The common changes of sensible and latent heat fluxes make the underlying surface of “water networks” show a warming effect, and the warm lake effect of the CBLs is more obvious. The diurnal variation of lake effect on the local atmospheric environment is consistent with the results of many previous studies [8,51]. However, different types of dense “water networks” exhibit different effects in the THB. The DSLs show a stronger cold effect during the day, while the CBLs present a stronger warm effect at night. Both are important reasons for the uneven distribution of the influences on the PM_2.5_ concentrations from the underlying surface of “water networks” in the THB.

**Figure 8:**
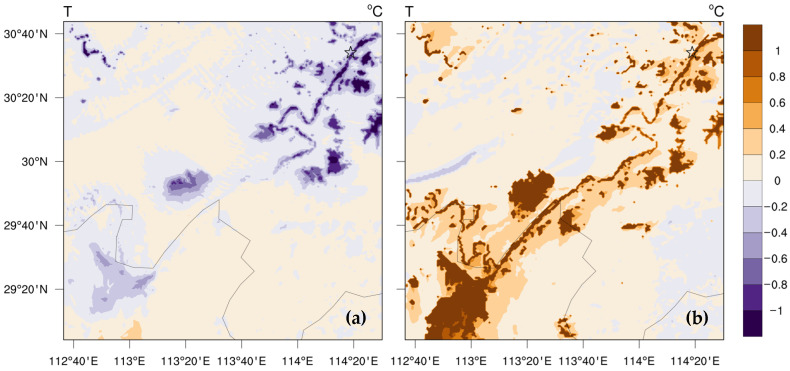

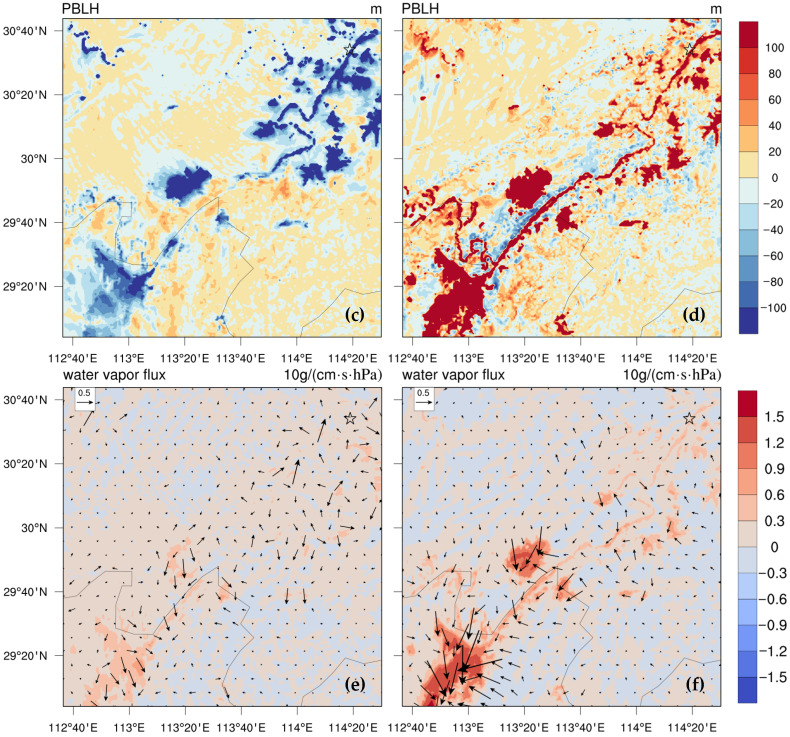
Spatial differences of (**a**,**c**,**e**) daytime (14:00 local time) and (**b**,**d**,**f**) nighttime (02:00 local time) (**a**,**b**) 2 m temperature, (**c**,**d**) atmospheric boundary layer height, and (**e**,**f**) water vapor flux transport in the horizontal direction between the lake experiment and no-lake experiment during the air pollution process from 19–22 January 2018.

The underlying surface of dense “water networks” in the THB obviously regulates the local thermal conditions, causing the changes in the structure of atmospheric boundary layer, which plays a crucial role in the distribution and diffusion of atmospheric particulate matter [8]. Figure 8 shows the spatial differences of daytime and nighttime 2 m temperature, atmospheric boundary layer height, and water vapor flux transport in the horizontal direction between the lake experiment and no-lake experiment. Figure S8 shows the variables in the lake experiment simulation. In the daytime, the cold lake effect of the DSLs is stronger (Figure 8a), which cools the atmospheric environment near the surface by 0.23 °C, and the vertical influence range reaches up to 1 km (Figure S9). As a result, the atmospheric stratification tends to be stable, and the height of the atmospheric boundary layer in the surrounding area of the lakes decreases remarkably (Figure 8c). The diurnal variation of the planetary boundary layer height is consistent with the diurnal variation of air temperature (Figure 7), especially in the DSL area; the height of local atmospheric boundary layer decreases by more than 100 m, which favors the accumulation of air pollutants. At night, the underlying surface of “water networks” shows a warm lake effect (Figure 8b), which causes an unstable and thicker atmospheric boundary layer (Figure 8d), indicating that the boundary layer changes over the water network are dominated by thermal effects, affecting the spatial and temporal variations of local PM_2.5_ over the “water network”. Especially in the CBL area, the stronger warm lake effect increases the temperature near the surface by 0.36 °C, and the vertical influence range reaches up to 500 m (Figure S9). The uplift of the local boundary layer even exceeds 100 m in local area (Figure 8d), which is favorable for the diffusion and alleviation of local pollution. The cold (warm) lake effect near the surface during the day (night) leads the atmospheric boundary layer to be more stabilized (unstable) and decreases (increases) the boundary layer height, which is conducive to the accumulation (diffusion) of local PM_2.5_ pollution.

The regulating effect of the underlying surface of “water networks” on local temperature causes the temperature differences between the lake surface and surrounding land area, which triggers local atmospheric circulation. During the day, the cold lake effect promotes the divergence of the local wind field and water vapor flux (Figure 8e). The smooth lake surface strengthens the local wind speed, which facilitates the diffusion and alleviation of local air pollution. The warm lake effect at night promotes the local convergence over the underlying surface of “water networks” (Figure 8f). Hence, the water vapor flux transported from the surrounding area is enhanced. The precursor and water vapor transport in surrounding source regions may aggravate the secondary generation and accumulation of PM_2.5_ particles.

In summary, the “water network” regulates the local air temperature by influencing the sensible and latent heat fluxes, which therefore changes the energy between the surface and atmosphere and the structure of the atmospheric boundary layer. Moreover, the local atmospheric circulation formed by the lake–land thermal difference drives the transport of air pollutants and water vapor from the surrounding source regions and further influences the spatiotemporal distribution of pollutants by affecting the physical processes in the atmospheric boundary layer and the generation of secondary aerosols.

Table 2 shows the correlations between the difference of PM_2.5_ concentration and the difference of meteorological elements between the lake experiment and no-lake experiment during the simulation period (312 samples during 11–24 January 2018). It is found that the PM_2.5_ changes induced by the underlying surface of “water networks” are significantly correlated with the changes in meteorological elements, proving that the atmospheric boundary layer over the underlying surface of dense “water networks” in the THB significantly affects the variation of regional PM_2.5_ concentration.

However, a problem to be further explored is which factor plays the dominant role under the joint action of these factors. Multiple linear regression is adopted to quantify the contribution of each factor to the changes in the dependent variables. The differences in the meteorological factors between the lake experiment and no-lake experiment are taken as independent variables, including sensible heat flux ($x_{1}$), latent heat flux ($x_{2}$), 2-m air temperature ($x_{3}$), 2-m relative humidity ($x_{4}$), atmospheric boundary layer height ($x_{5}$), and 10-m wind speed ($x_{6}$). With the difference of PM_2.5_ concentration between the lake experiment and no-lake experiment as the dependent variable (y), a set of standardized multiple linear regression equations are established, as follows:

(7) $$y=-0.2988x_{1}+0.6379x_{2}-0.8500x_{3}+0.3232x_{4}-0.0190x_{5}+0.0601x_{6},$$

(8) $$y=-0.2702x_{1}+0.3668x_{2}-0.8317x_{3}+0.0894x_{4}-0.0364x_{5}-0.0759x_{6},$$

Equation (7) is the standardized multiple linear regression equation averaged over the CBLs, and Equation (8) is for the DSLs. The correlation coefficients between the fitting results and dependent variables (y) in Equations (7) and (8) are 0.77 and 0.76, respectively, and both pass the *t*-test at the 0.01 significance level. The positive (negative) coefficients in the equation indicate that a certain independent variable exerts the same (opposite) effect on the dependent variable as the joint influence of all variables.

The interaction between thermal factors and water vapor over the underlying surface of “water networks” is an important mechanism affecting the changes of the atmospheric boundary layer and the aerosol physicochemical processes. Equations (7) and (8) reveal that the changes of sensible heat flux ($x_{1}$) and 2-m air temperature ($x_{3}$), as thermal factors, pose negative effects on the change of PM_2.5_ concentration between the presence and absence of lakes, that is, the warm (cold) lake effect promotes the decrease (increase) in PM_2.5_ concentration. The increases in latent heat flux ($x_{2}$) and 2-m relative humidity ($x_{4}$) over the underlying surface of “water networks” contribute positively to the increase in local PM_2.5_ concentration. The latent heat flux represents the vertical transport of water vapor in water–heat exchange between the “water network” and atmosphere. The lake acts a long-term water vapor source in the lower atmosphere, and the higher latent heat flux and relative humidity have obvious humidification effects on the atmosphere. The water vapor is an important factor affecting the hygroscopic growth of aerosols, which can promote the liquid-phase heterogeneous reaction and accelerate the gas-particle transformation, thus accelerating the generation of secondary aerosols. Hence, the PM_2.5_ concentration increases [52,53]. The height of the atmospheric boundary layer has a negative effect on the PM_2.5_ concentration, where the increase in the height is beneficial to the decrease in PM_2.5_ concentration. In addition, the change of wind speed over the underlying surface of “water networks” of the CBLs positively affects the change of PM_2.5_ concentration, which may be due to the transports of pollutants or water vapor by the wind. However, for the underlying surface of “water networks” of the DSLs, the change of wind speed poses a negative impact, showing the removal effect of enhanced wind speed on PM_2.5_ particles over the smooth lake surface.

The standardized regression coefficients were used to estimate the relative contributions of thermal, water vapor, and dynamic factors to the change in PM_2.5_ concentration over the underlying surface of “water networks” of the CBLs and DSLs (Table 3). For the CBLs, the relative contribution rates of thermal factors (sensible heat flux and air temperature) and water vapor-related factors (latent heat flux and relative humidity) are 52.48% and 43.91%, respectively. These two factors jointly dominate the change in PM_2.5_ concentrations, while the relative contribution rate of dynamic factors (atmospheric boundary layer height and wind speed) is only 3.62%.

For the DSLs, the thermal factors dominate the change in PM_2.5_ concentrations, which contributes 65.96% to the total. The relative contribution rate of water vapor-related factors is 27.31%, indicating that the water vapor-related factors contribute obviously less to the PM_2.5_ over the DSLs than over the CBLs. The relative contribution rate of dynamic factors is 6.72%. These results reflect the difference of lake effect over different types of lakes.

In summary, the thermal and water vapor-related factors are the key meteorological factors through which the underlying surface of “water networks” affects the atmospheric environment of the THB. The thermal factors mainly affect the structure of the atmospheric boundary layer, and the water vapor-related factors mainly contribute to the humidification effect on the atmosphere. The warming and humidification effects of the “water networks” contribute positively and negatively to the “purification” of air pollution, respectively.

As reported by previous studies, lake-land breeze triggers the distinctive wind patterns between daytime and nighttime, which change both the dispersion and transport process of PM_2.5_ [20,21]. Although the intensity of lake breeze in our study on heavy PM_2.5_ pollution is much weaker compared with a typical sea breeze in previous reports, the thermal effects of lake breeze in inland lakes on the local meteorology could significantly affect the change of PM_2.5_ concentrations in heavy PM_2.5_ pollution. This may be related with the thermal and dynamic effects as affected by the density and area of water networks under different environment and climate backgrounds. 

## 4. Conclusions

This study conducted a set of experiments on the impact of dense “water networks” on a wintertime PM_2.5_ pollution event in the THB. By comparing the simulation results of a lake experiment and no-lake experiment, the influence of the underlying surface of dense “water networks” in the THB on the variations of PM_2.5_ concentrations were investigated. Moreover, the key thermal factors and water vapor-related factors in the changes of PM_2.5_ concentrations in the atmospheric boundary layer were evaluated. The main conclusions are as follows:

The underlying surface of dense “water networks” in the THB leads to the change in PM_2.5_ concentrations in association with the density and area of lake groups. The CBLs reduce the local PM_2.5_ concentrations by 4.90–17.68% during the day and night. The influence of the lake on PM_2.5_ concentrations reaches up to 1.5 km in the vertical direction, and the PM_2.5_ concentration can be reduced by 9.60–13.11% from the ground up to 500 m. On the contrary, the ability of the DSLs to alleviate PM_2.5_ pollution is weaker. The influences of the DSLs on the average PM_2.5_ concentrations over the lake and surrounding areas range from −5.63% to 1.56%. Moreover, the PM_2.5_ pollution may even be aggravated during the daytime.

The CBLs show a stronger warm source effect at night, while the DSLs show a stronger cold source effect during the day, which are important reasons for the unevenly-distributed influences on regional PM_2.5_ concentration by the underlying surface of “water networks” in the THB. The dense “water network” in the THB regulates local air temperature by influencing the changes in sensible and latent heat fluxes. The changes in the land-air energy budget change the structure of atmospheric boundary layer, and the warm (cold) lake effect promotes the decrease (increase) in PM_2.5_ concentration. Meanwhile, the local atmospheric circulation formed by the lake-land thermal difference also drives the transport of air pollutants and water vapor from the surrounding source areas, which further affects the spatiotemporal distribution of PM_2.5_ concentration by influencing the physical process in the atmospheric boundary layer and the generation of secondary aerosols.

Thermal factors and water vapor-related factors are the key meteorological factors affecting the atmospheric environment of the THB over the underlying surface of the “water network”. The warming and humidification effects of the “water network” contribute positively and negatively to the “purification” of air pollution, respectively. For the CBLs, the relative contribution rates of thermal factors and water vapor–related factors are 52.48% and 43.91%, respectively. The two factors jointly dominate the change in PM_2.5_ concentrations. For the DSLs, the thermal factors contribute 65.96% of the total, which dominate the change in PM_2.5_ concentrations, while the relative contribution rate of water vapor-related factors is 27.31%. This indicates that the contribution of water vapor-related factors over the CBLs is stronger.

In order to fully understand the potential impact of the underlying “water network” surface on air pollution, long-term studies should be carried out, with the fine meteorological and environmental measurements involving air-surface exchanges of energy and water as well as physical and chemical processes of air pollution and more comprehensive simulations of lake breeze and local air pollution under different scenarios.

## Figures and Tables

**Table 1:** Statistical evaluation of simulation results at Wuhan and Yueyang stations from 20:00 11 January to 20:00 24 January 2018.

Station	Variables	R	RMSE	MB	GE	MFB (%)	MFE (%)
Wuhan	2-m temperature (°C)	0.78 **	3.88	3.11	3.25	47.33	49.42
	2-m relative humidity (%)	0.71 **	26.43	−23.34	23.37	−20.94	20.97
	10-m wind speed (m/s)	0.57 **	1.28	0.56	1.03	44.83	59.11
	10-m wind direction (°)	0.27**	139.65	−5.68	83.88	9.20	39.95
	Surface air pressure (hPa)	0.99 **	0.88	−0.46	0.71	−0.03	0.05
	Precipitation rate (mm/h)	0.60 **	0.14	0.00	0.04	/	/
	Near-surface PM_25_ (μg/m^3^)	0.56 **	56.34	28.68	38.99	16.41	22.03
Yueyang	2-m temperature (°C)	0.80 **	3.58	3.32	3.32	33.26	33.29
	2-m relative humidity (%)	0.82 **	16.44	−12.69	14.18	−10.14	12.14
	10-m wind speed (m/s)	0.64 **	2.41	1.88	2.05	50.45	55.39
	10-m wind direction (°)	0.41**	117.98	−8.36	71.31	9.34	42.92
	Surface air pressure (hPa)	0.99 **	1.49	1.30	1.32	0.09	0.09
	Precipitation rate (mm/h)	0.70 **	0.21	−0.02	0.09	/	/
	Near-surface PM_25_ (μg/m^3^)	0.31 **	42.70	1.86	35.63	5.15	26.78

Note: ** indicates that the values have passed the significance test at the 0.01 significance level.

**Table 2:** Correlation coefficients of PM_2.5_ concentration difference with influencing factor difference between lake experiment and no-lake experiment.

Variables	CBL	DSL
Sensible heat flux	−0.35 **	−0.47 **
Latent heat flux	−0.37 **	−0.57 **
2-m temperature	−0.64 **	−0.75 **
2-m relative humidity	0.54 **	−0.08
Atmospheric boundary layer height	−0.40 **	−0.52 **
10-m wind speed	0.01	−0.40 **

Note: ** indicates that the values have passed the significance test at 0.01 significance level.

**Table 3:** Contributions of different factors affected by the underlying surface of “water networks” to the change of PM_2.5_ concentrations.

Variables	CBLs	DSLs
Sensible heat flux	13.65%	16.17%
Latent heat flux	29.14%	21.96%
2-m temperature	38.83%	49.79%
2-m relative humidity	14.77%	5.35%
Atmospheric boundary layer height	0.87%	2.18%
10-m wind speed	2.75%	4.54%

## Data Availability

Data used in this paper may be provided by Yan Zhu (zhuyan620@163.com) upon request.

## Supplementary Materials

The following supporting information can be downloaded at: https://www.mdpi.com/article/10.3390/toxics11020169/s1, Figure S1: The distribution of (**a**) land and water, (**b**) simulated water vapor flux (QFX), and (**c**) raster pixel image of CCD2 Level2 on HJ1A Satellite (horizontal resolution of 30 m) in the modeling domain d03. The small box indicates the DSLs, the large box indicates the CBLs; Figure S2: The spatial distributions of (**a**) biogenic VOC emissions from MEGAN, and (**b**) NMVOC emissions from MEIC in the modeling domain d01 averaged for the simulation period; Figure S3: Spatial distributions of (left panel) observed and (right panel) simulated near–surface PM_2.5_ concentrations (color dots (left panel), color contours (right panel), μg/m^3^), 10-m wind vectors (m/s) and sea level pressure (hPa) at 00:00 (UTC) during 14–24 January, 2018. Blue lines indicate sea level air pressure. Blue area denotes the Pacific Ocean in the left panels; Figure S4: Time variations of simulated near-surface PM_2.5_ concentrations at Wuhan station during the simulation period. Orange line shows the threshold of heavy pollution level; Figure S5: Diurnal variations of PM_2.5_ concentrations averaged over CBLs and DSLs during air pollution process from 19–22 January 2018 in the lake experiment of simulation; Figure S6: Vertical distribution of daytime (14:00 in local time) and nighttime (02:00 in local time) PM_2.5_ concentrations below the height of 2 km in the atmosphere averaged over CBLs and DSLs during air pollution process from 19 to 22 January 2018 in the lake experiment of simulation; Figure S7: Diurnal variations (hourly Time) of (**a**) sensible heat flux (HFX, W/m^2^), (**b**) latent heat flux (LH, W/m^2^), (**c**) 2-m air temperature (T2, °C), and (**d**) planetary boundary layer height (PBLH, m) averaged over CBLs and DSLs during air pollution process over January 19–22, 2018 in the lake experiment of simulation; Figure S8: Spatial distributions of (**a**,**b**) 2-m temperature, (c, d) atmospheric boundary layer height, and (**e**,**f**) water vapor flux transport in the horizontal direction averaged in the (**a**,**c**,**e**) daytime (14:00 in local time) and (**b**,**d**,**f**) nighttime (02:00 in local time) from the simulations of the lake experiment during the pollution process from 19 to 22 January 2018; Figure S9: Vertical distribution of air temperature differences (Temperature) between the simulations of lake experiment and no-lake experiment averaged for CBLs and DSLs (Height) below 2 km in the atmosphere during the air pollution process from 19–22 January 2018; Table S1: Statistical evaluation of 10-m wind direction at Wuhan and Yueyang station from 20:00 11 January to 20:00 24 January 2018.
