# Supplementary material for: Mitigation Effect of Dense “Water Network” on Heavy PM2.5 Pollution: A Case Model of the Twain-Hu Basin, Central China
Source: Toxics. 2023 Feb 10;11(2):169. doi: 10.3390/toxics11020169 (PMC9966530; doi:10.3390/toxics11020169)
Supplement: Supplementary file 1 [file toxics-11-00169-s001.zip › toxics-2170520-supplementary.pdf]

Mitigation Effect of Dense "Water Network" on Heavy $PM_{2.5}$ Pollution: A case Model of the Twain-Hu Basin, Central China

Yan Zhu1,2，Yongqing Bai2*，Jie Xiong2*，Tianliang Zhao3，Jiaping Xu4，Yue Zhou2，Kai Meng5，Chengzhen Meng5，Xiaoyun Sun3，Weiyang Hu6

1. Hubei Meteorological Service Center, Wuhan 430205 China
2. China Meteorological Administration Basin Heavy Rainfall Key Laboratory/Hubei Key Laboratory for Heavy Rain Monitoring and Warning Research, Institute of Heavy Rain, China Meteorological Administration, Wuhan 430205, China
3. Key Laboratory for Aerosol-Cloud-Precipitation of China Meteorological Administration, Collaborative Innovation Center on Forecast and Evaluation of Meteorological Disasters, Nanjing University of Information Science and Technology, Nanjing 210044, China
4. Jiangsu Climate Center, Nanjing 210044, China
5. Key Laboratory of Meteorology and Ecological Environment of Hebei Province, Hebei Provincial Institute of Meteorological Sciences, Shijiazhuang 050021, China
6. State Key Laboratory of Pollution Control and Resource Reuse and School of the Environment, Nanjing University, Nanjing 210023, China

* Correspondence: baiyq@whihr.com.cn (Y.B.); xiongjie@whihr.com.cn (J.X.)

Supplementary Materials

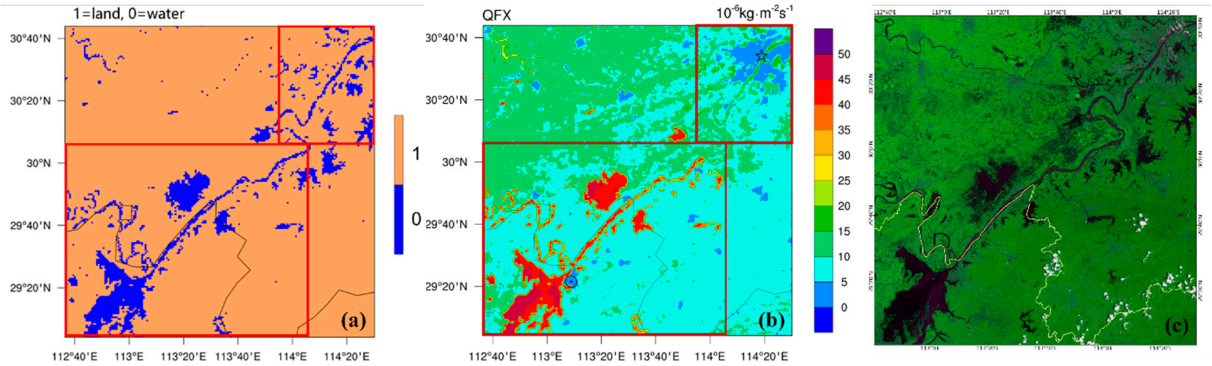

Figure S1. The distribution of (a) land and water, (b) simulated water vapor flux (QFX), and (c) raster pixel image of CCD2 Level2 on HJ1A Satellite (horizontal resolution of 30 m) in the modeling domain d03. The small box indicates the DSLs, the large box indicates the CBLs.

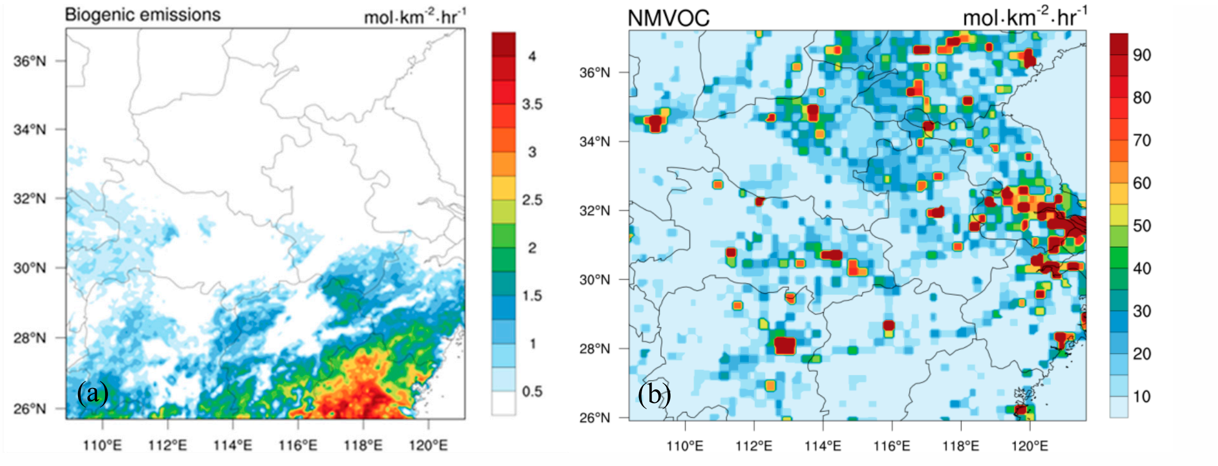

Figure S2. The spatial distributions of (a) biogenic VOC emissions from MEGAN, and (b) NMVOC emissions from MEIC in the modeling domain d01 averaged for the simulation period.

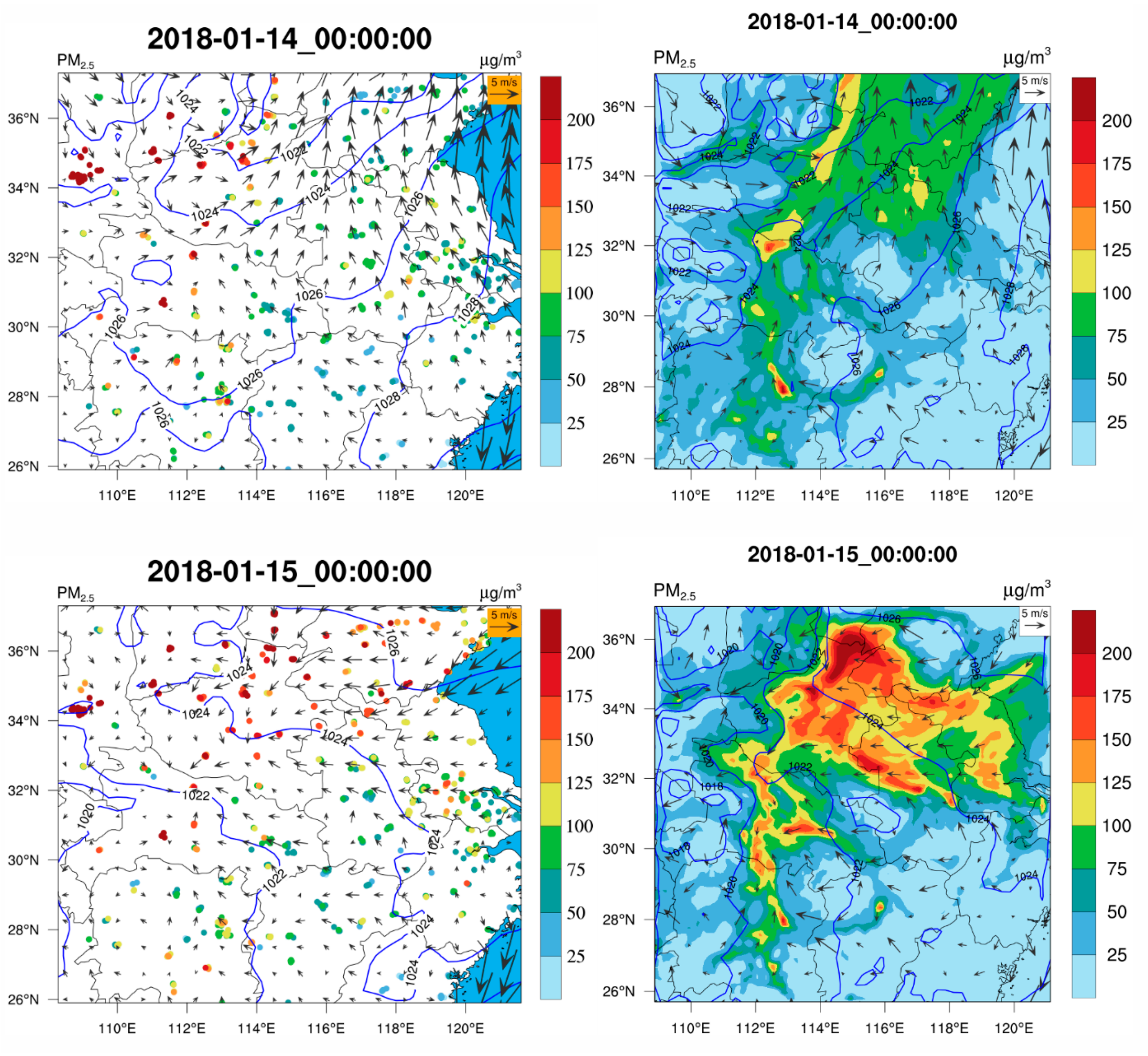

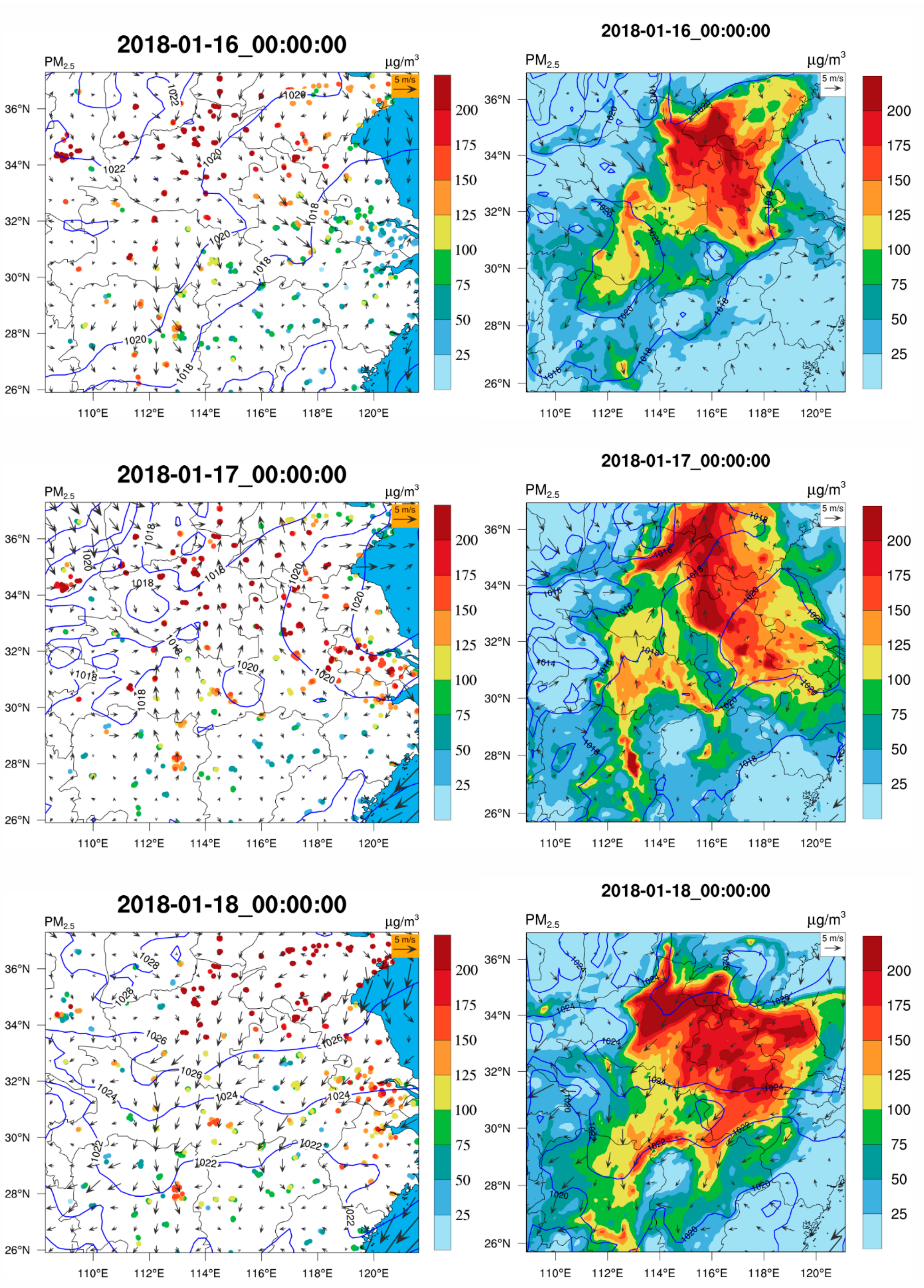
2018-01-16_00:00:00
PM2.5
µg/m3
5 m/s
200
175
150
125
100
75
50
25
36°N
34°N
32°N
30°N
28°N
26°N
110°E
112°E
114°E
116°E
118°E
120°E
2018-01-16_00:00:00
2018-01-17_00:00:00
2018-01-17_00:00:00
2018-01-18_00:00:00
2018-01-18_00:00:00

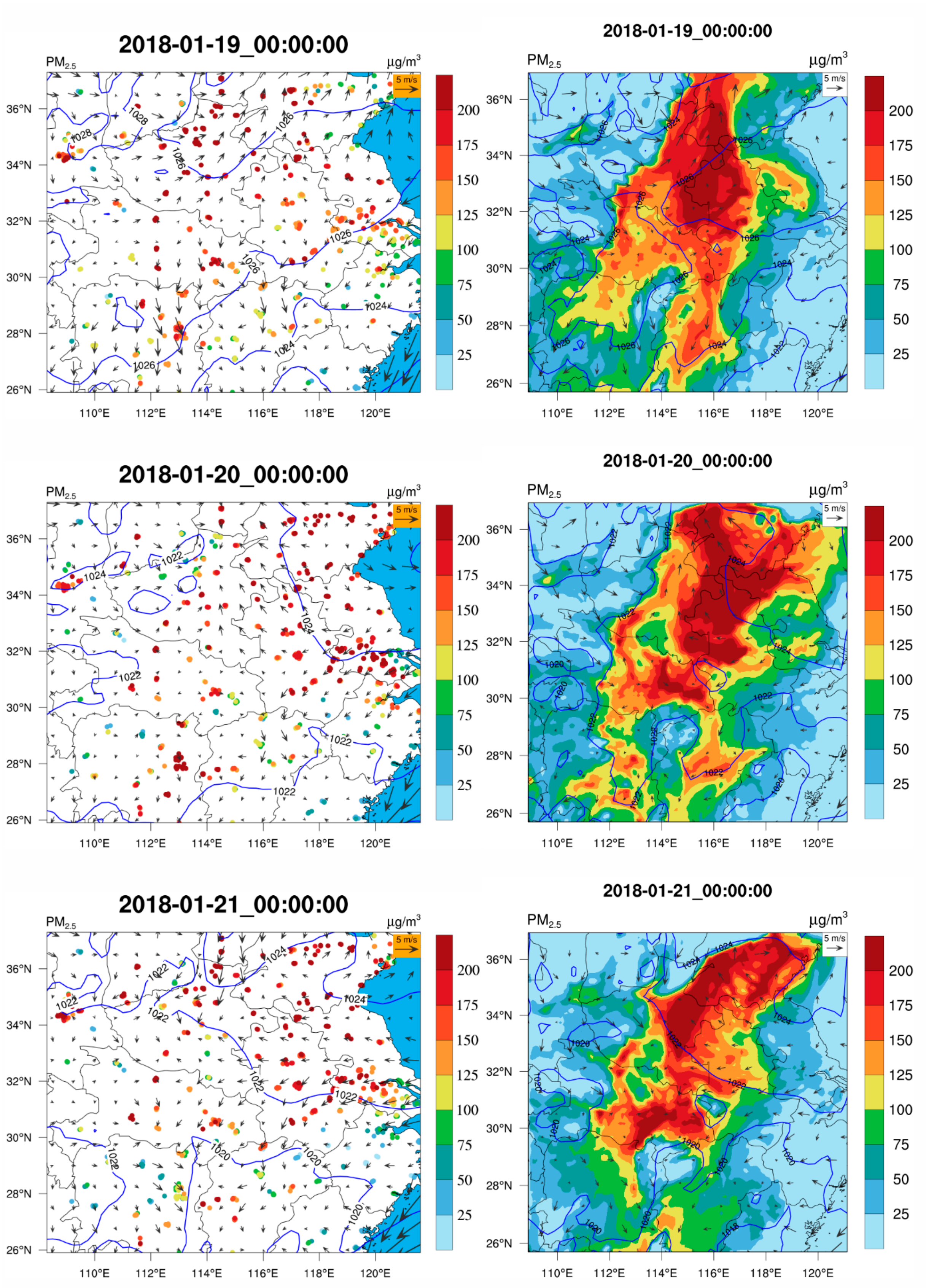
2018-01-19_00:00:00
PM2.5
µg/m3
5 m/s
200
175
150
125
100
75
50
25
36°N
34°N
32°N
30°N
28°N
26°N
110°E
112°E
114°E
116°E
118°E
120°E
2018-01-20_00:00:00
2018-01-21_00:00:00

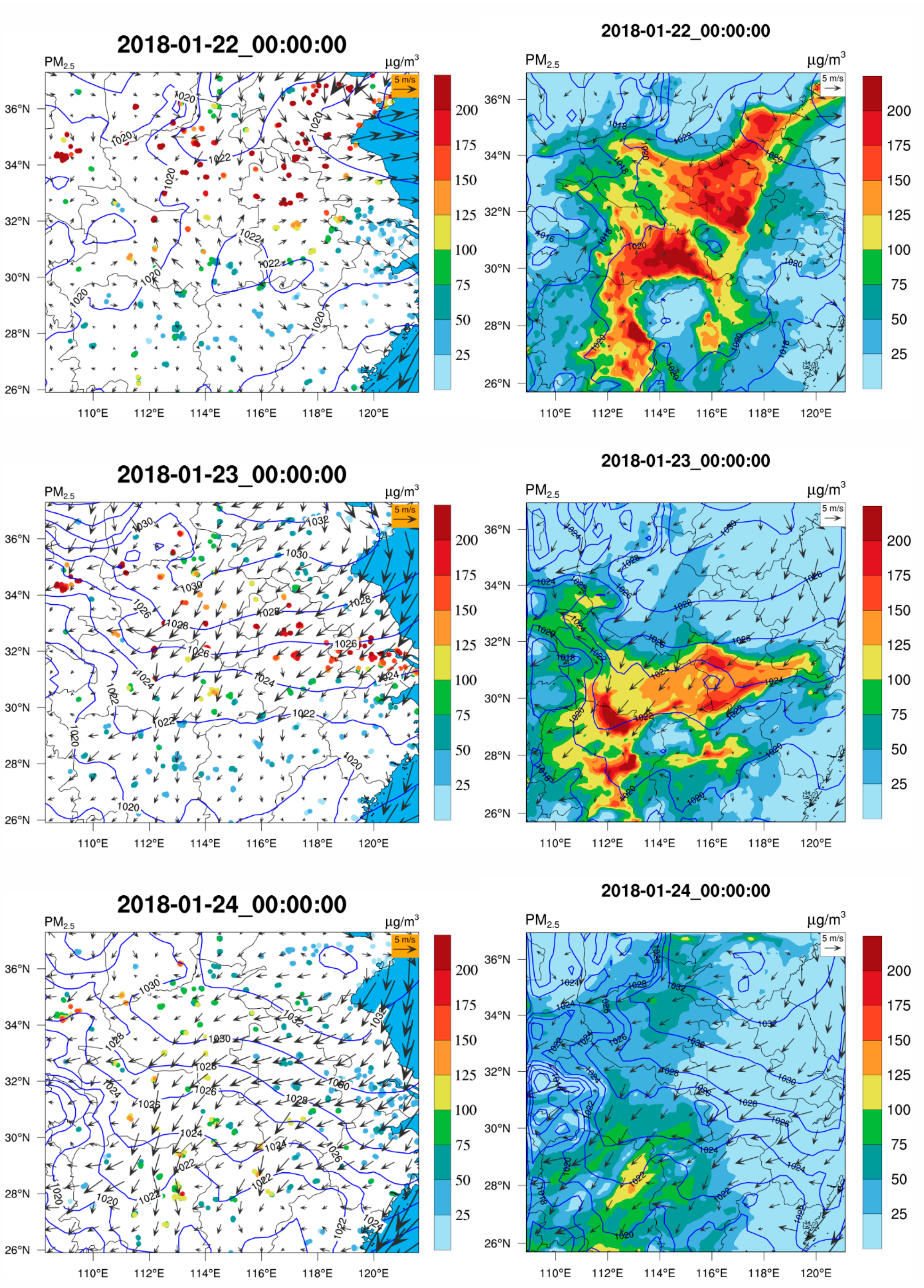

Figure S3. Spatial distributions of (left panel) observed and (right panel) simulated near-surface $PM_{2.5}$ concentrations (color dots (left panel), color contours (right panel), μg/m^3), 10-m wind vectors (m/s) and sea level pressure (hPa) at 00:00 (UTC) during January 14–24, 2018. Blue lines indicate sea level air pressure. Blue area denotes the Pacific Ocean in the left panels.

Table S1. Statistical evaluation of 10-m wind direction at Wuhan and Yueyang station from 20:00 January 11 to 20:00 January 24, 2018.

	Direction diff (°)	
	mean	standard deviation
Wuhan	0.37	72.39
Yueyang	–3.43	59.64

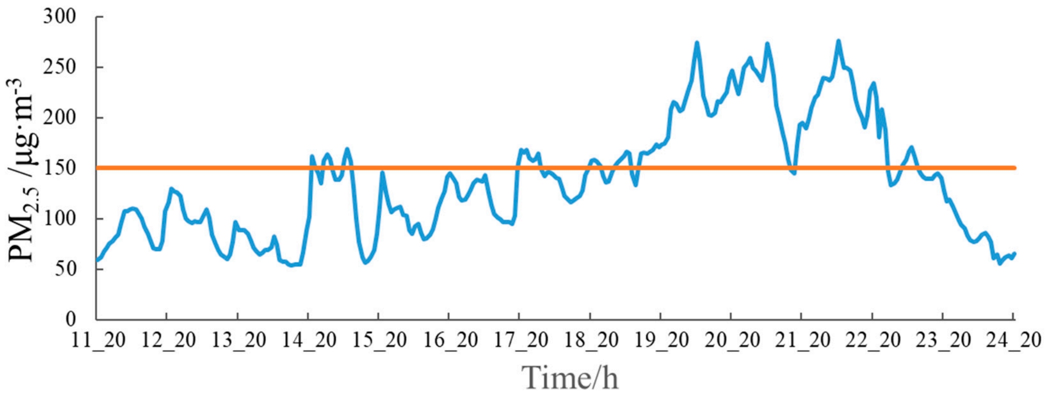

Figure S4. Time variations of simulated near-surface $PM_{2.5}$ concentrations at Wuhan station during the simulation period. Orange line shows the threshold of heavy pollution level.

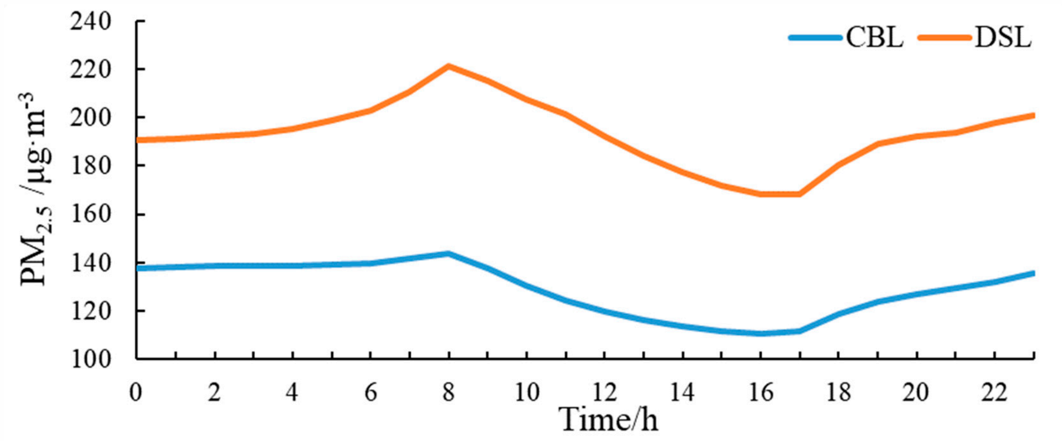

Figure S5. Diurnal variations of $PM_{2.5}$ concentrations averaged over CBLs and DSLs during air pollution process from January 19–22, 2018 in the lake experiment of simulation.

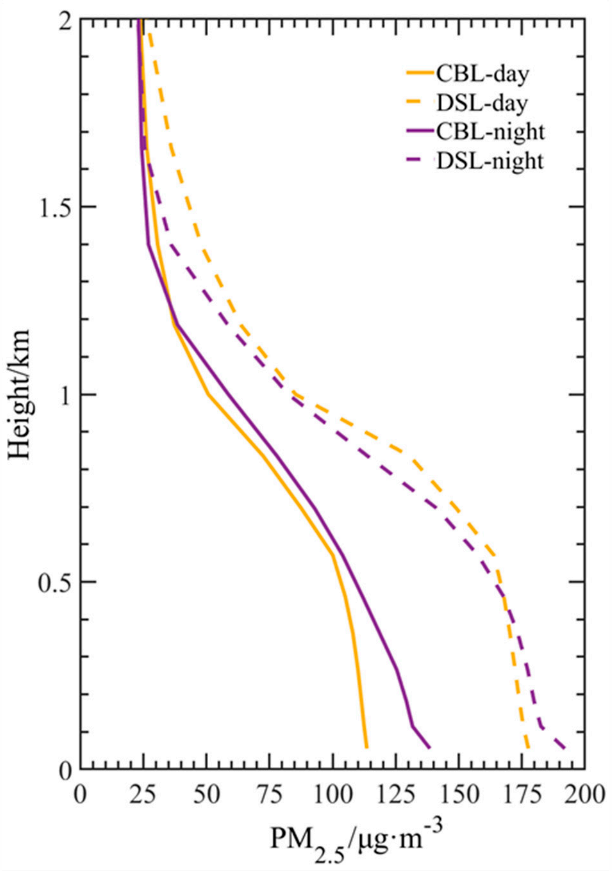

Figure S6. Vertical distribution of daytime (14:00 in local time) and nighttime (02:00 in local time) $PM_{2.5}$ concentrations below the height of 2 km in the atmosphere averaged over CBLs and DSLs during air pollution process from January 19 to 22, 2018 in the lake experiment of simulation.

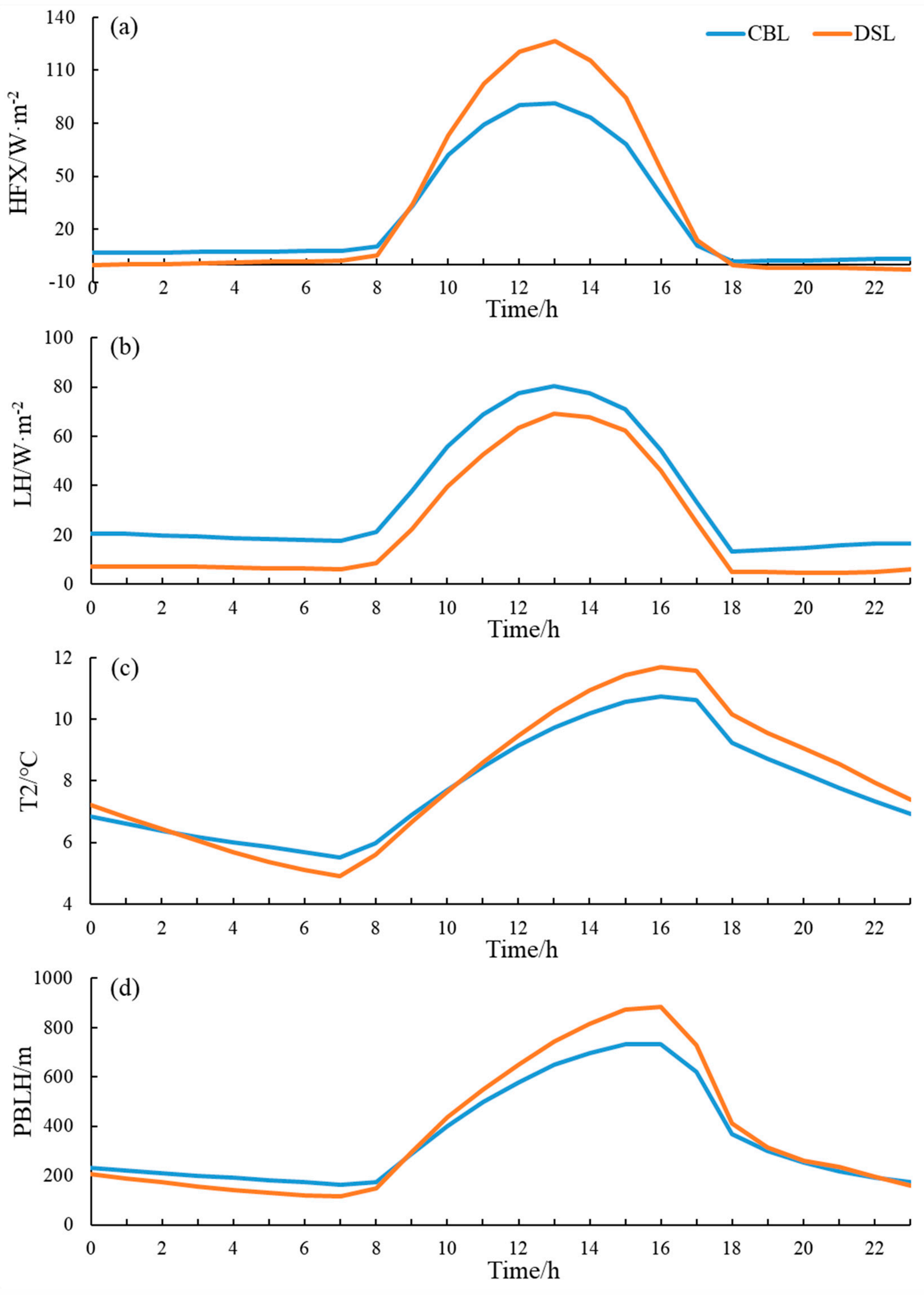

Figure S7. Diurnal variations (hourly Time) of (a) sensible heat flux (HFX, W·m^{-2}), (b) latent heat flux (LH, W·m^{-2}), (c) 2-m air temperature (T2, °C), and (d) planetary boundary layer height (PBLH, m) averaged over CBLs and DSLs during air pollution process over January 19–22, 2018 in the lake experiment of simulation.

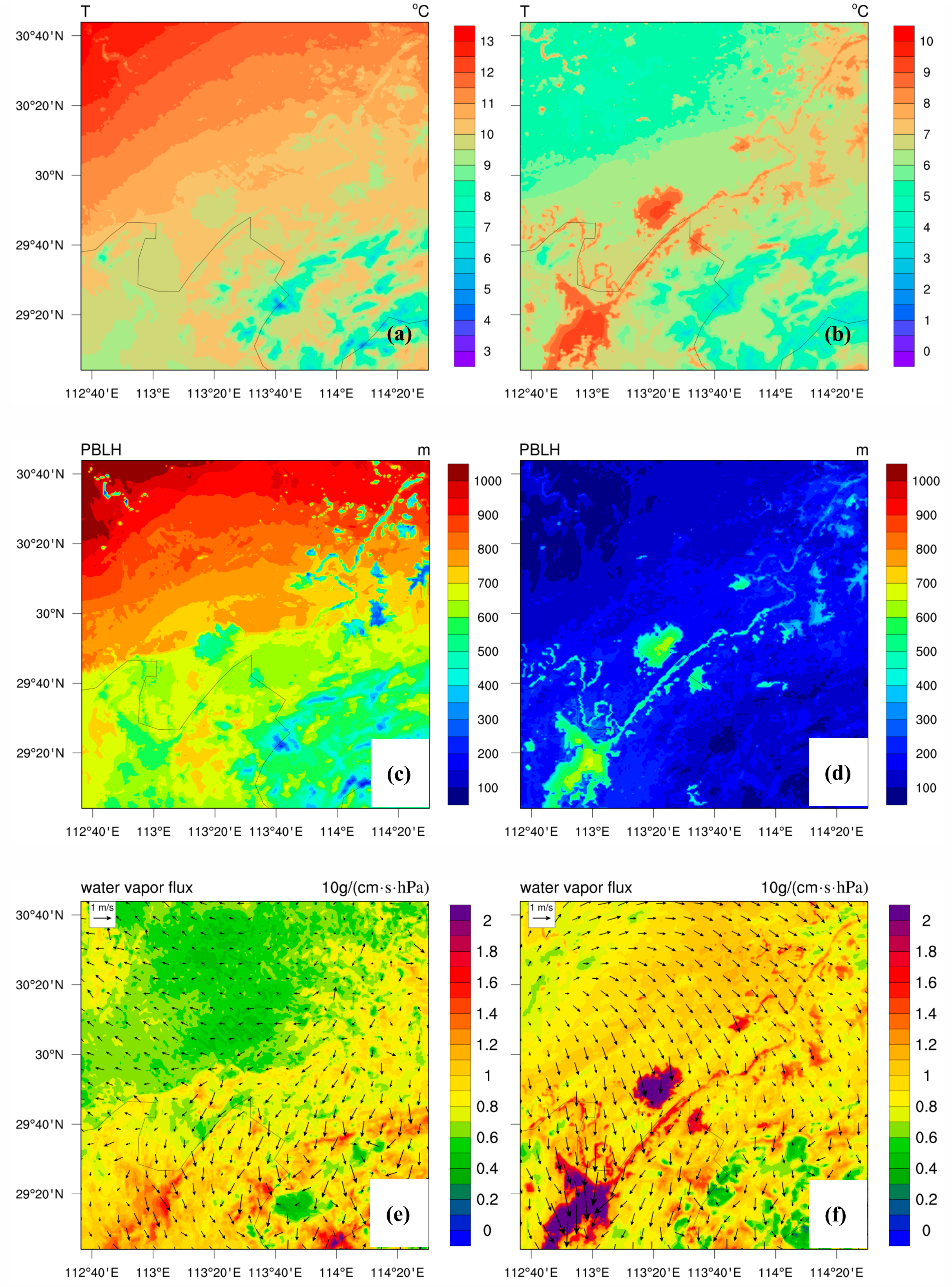

Figure S8. Spatial distributions of (a, b) 2-m temperature, (c, d) atmospheric boundary layer height, and (e, f) water vapor flux transport in the horizontal direction averaged in the (a, c, e) daytime (14:00 in local time) and (b, d, f) nighttime (02:00 in local time) from the simulations of the lake experiment during the pollution process from January 19 to 22, 2018.

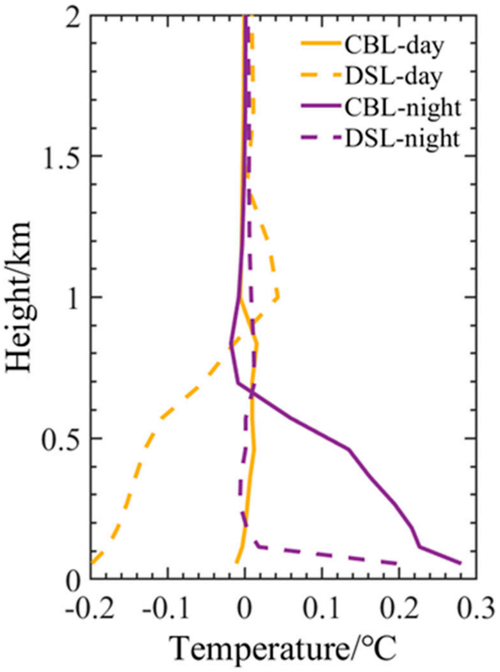

Figure S9. Vertical distribution of air temperature differences (Temperature) between the simulations of lake experiment and no-lake experiment averaged for CBLs and DSLs (Height) below 2 km in the atmosphere during the air pollution process from January 19─22, 2018.
